# Single-nuclei RNA-seq on human retinal tissue provides improved transcriptome profiling

**DOI:** 10.1038/s41467-019-12917-9

**Published:** 2019-12-17

**Authors:** Qingnan Liang, Rachayata Dharmat, Leah Owen, Akbar Shakoor, Yumei Li, Sangbae Kim, Albert Vitale, Ivana Kim, Denise Morgan, Shaoheng Liang, Nathaniel Wu, Ken Chen, Margaret M. DeAngelis, Rui Chen

**Affiliations:** 1 HGSC, Department of Molecular and Human Genetics, Baylor College of Medicine, Houston, TX 77030 USA; 2 Department of Molecular and Human Genetics, Baylor College of Medicine, Houston, 77030 TX USA; 3 Verna and Marrs McLean Department of Biochemistry and Molecular Biology, Baylor College of Medicine, Houston, TX 77030 USA; 4 Department of Ophthalmology and Visual Sciences, University of Utah School of Medicine, Salt Lake City, UT 84132 USA; 5 Department of Pharmacotherapy, the College of Pharmacy, University of Utah, Salt Lake City, UT 84132 USA; 6 Department of Bioinformatics and Computational Biology, The University of Texas MD Anderson Cancer Center, Houston, TX 77030 USA; 7 Department of Population Health Sciences, University of Utah School of Medicine, Salt Lake City, UT 84132 USA; 8 Present Address: St. Jude Children’s Research Hospital, 262 Danny Thomas Pl, Memphis, TN 38105 USA

**Keywords:** Bioinformatics, Gene expression analysis, Genetics, Gene expression, Sequencing

## Abstract

Single-cell RNA-seq is a powerful tool in decoding the heterogeneity in complex tissues by generating transcriptomic profiles of the individual cell. Here, we report a single-nuclei RNA-seq (snRNA-seq) transcriptomic study on human retinal tissue, which is composed of multiple cell types with distinct functions. Six samples from three healthy donors are profiled and high-quality RNA-seq data is obtained for 5873 single nuclei. All major retinal cell types are observed and marker genes for each cell type are identified. The gene expression of the macular and peripheral retina is compared to each other at cell-type level. Furthermore, our dataset shows an improved power for prioritizing genes associated with human retinal diseases compared to both mouse single-cell RNA-seq and human bulk RNA-seq results. In conclusion, we demonstrate that obtaining single cell transcriptomes from human frozen tissues can provide insight missed by either human bulk RNA-seq or animal models.

## Abstract (web summary)

The retina is a heterogeneous tissue composed of multiple cell types. Via single-nuclei RNA sequencing on human neural retinal tissue, the authors characterise the transcriptome profile for individual cell types in the human retina.

## Introduction

The retina is a heterogeneous tissue and is composed of multiple neuronal and non-neuronal cell types^1^. In the human retina, there is an ordered array of ~70 different cell types across five major neuron classes: photoreceptors (rods and cones), retinal ganglion cells (RGCs), horizontal cells (HC), bipolar cells (BC), amacrine cells (AC), and a non-neuronal Müller glial cell (MG), each playing a unique role in processing visual signals^1,2^. The transcriptome of the human retina has been reported using bulk tissue RNA-seq^3,4^, and the overall gene expression profiles from different retinal regions (macular and peripheral region) were compared to each other^5^. These studies provided general transcriptomic information of the retina as a whole tissue, but they could not reveal the entirety of the retina’s complexity because it lacks individual cell-type resolution. In addition, transcriptomic studies of selected cell types of the human and primate retina were performed but were not sufficient in obtaining the complete profile of all major cell types^6,7^. The profiles of individual cell types, particularly rare types that account for a small portion of the retina, will offer important insights to the scientific knowledge of biology and disease. For example, particular cell types, such as the cone cells in the cone-rod dystrophy (CRD) and the retinal ganglion cells in glaucoma, are the first targets of the diseases^8,9^. However, both cell types are found in low proportions in the human retina and their transcriptome profiles have not been well studied. Given the great benefit of obtaining the transcriptome at the individual cell type or at the individual cell level, single-cell RNA-seq on human retina tissue is an area of great interest.

Recently, single-cell RNA-seq studies have been performed on mouse retina, both within the whole retina^10^ and within specifically sorted cell types^11,12^. These studies provided unprecedentedly high-resolution transcriptomic data of each cell types and allowed for novel cell subtype discovery. However, usage of mouse datasets could be limiting given the considerable differences between the human and mouse retina. For example, the mouse retina lacks the macula region of the retina that is found in humans and primates^13,14^, a structure that is essential for both high visual acuity and color vision perception in the retina. Mouse cone cells are also different from those of humans in their wavelength-sensitive opsin expression patterns. Thus, we propose that single-cell transcriptomic study on human retinal tissues will expand our knowledge of the retina and become a rich resource for related research, such as human disease-related studies.

Unlike model-organism-based studies, more factors are needed to be taken into consideration for human tissue studies in order to provide reliable results. One of the major concerns in profiling the transcriptome of post-mortem human tissues is RNA integrity. This issue worsens in single-cell-level studies because the dissociation of tissue occurs for an extended period of time after the retina is dissected. Thus, the dissociation itself could also lead to changes in transcription due to damage of the retina that might have occurred during the waiting period. Another concern is the health condition of the human tissue, since studies using multiple individuals with differing health conditions could potentially add complexity in proper interpretation of the data. Here, we report a single-cell transcriptomic study on healthy human retina tissues using snRNA-seq. We tackle the potential issues by two approaches. The first consisted of using snRNA-seq instead of single-cell RNA-seq (scRNA-seq) to profile tissues confirmed as healthy by a strict post-mortem phenotyping. The second approach involved snRNA-seq, where tissues were flash-frozen immediately after dissection to preserve RNA integrity, minimizing the variation due to differences in tissue dissociation conditions. With the extensive post-mortem phenotyping, we can ensure only healthy tissues are used in our study.

Using the snRNA-seq approach, a total of 5873 nuclei are profiled from both the peripheral and macular region of three frozen human donor retina samples. Through unsupervised clustering of the gene expression profiles, clusters corresponding to all seven major cell types in the human retina (rod, cone, MG, HC, AC, BC, and RGC) are identified. Differentially expressed genes from each cell type are obtained. We compare the gene expression profile between macular and peripheral region for each cell types. Significantly higher expression of mitochondrial-electron transport genes is found in macular rod cells compared to the expression of the peripheral ones, which may indicate that comparatively higher levels of oxidation stress exists in the macular, providing a potential explanation of higher vulnerability of macular rod cells during aging. In addition, as expected, compared to the published mouse single-cell data, the single-nuclei human data show stronger predictive power in prioritizing genes associated with human disease. Finally, we find that photoreceptor DEGs significantly enrich inherited retinal disease (IRD) genes, indicating that they can serve as a prioritization tool for novel disease gene discovery and cell-specific pathway analysis. Overall, our study reports a transcriptome profile of all major cell types of the human healthy retina at the resolution of the individual cell, which would serve as a rich resource for the scientific community.

## Results

### The generation of the snRNA-seq data of human retina

**Fig. 1:**
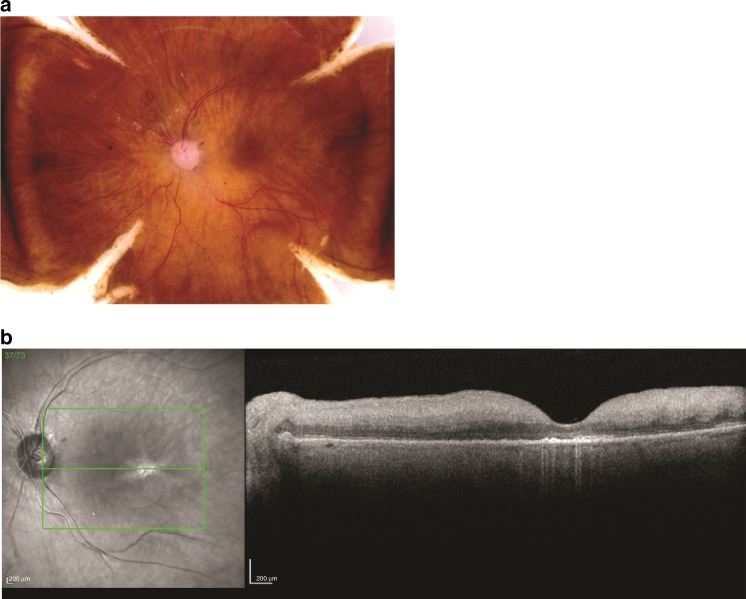
**Analogous color fundus and OCT images demonstrating normal findings for post-mortem eyes. a** Color fundus imaging of post-mortem retina showing a normal phenotype. **b** OCT image of post-mortem retina showing a normal phenotype.

To generate transcriptome profiles for human photoreceptor cells, retinae from three separate, healthy donors were obtained (*n* = 3 donors). All three donors were Caucasian, ages between 60 and 80 years old (Table 1), and were thoroughly examined as previously described in the methods. Figure 1 shows an example of the donor tissues used for this study. There was no visible pathogenic indication found, not even macular drusen, a hallmark of age-related macular degeneration commonly found in this age group, according to the fundus images^15^. Two-sample punches from each retina (*n* = 6), one from the macula region and the other from the peripheral region, were collected and subjected to single-cell nuclei RNA-Seq. After dispensing, nanowells were imaged and only wells with single nuclei were selected. cDNA library construction and sequencing were performed for a total of 6542 individual nuclei. Distribution of the number of nuclei from each sample is listed in Table 2. To exclude low-quality data, several QC steps were conducted (described in the methods). As a result, 669 nuclei were filtered out, leaving a total number of 5873 nuclei for downstream analysis. On average, 31,186 mapped reads were obtained per nucleus, with the median number of genes detected at 1044. To further evaluate the quality of snRNA-Seq data, bulk RNA-seq from the corresponding sample was performed. Good correlation between the bulk gene expression (FPKM, paired-end seq) and single-nuclei gene expression (average of normalized read count after transformation and 3′ end seq, see methods) was observed with positive correlation coefficient ranges from 0.66 to 0.71 (all genes with non-zero expression were used with gene numbers ranging from 12163 to 13657, among six samples. Spearman correlation coefficient was used).

**Table 1:** Medical information of the sample donors.

Donor number	Globe	Age	Sex	Race
12–887	OS (left eye)	78	F	Caucasian
13–0025	OD (right eye)	78	M	Caucasian
13–0347	OS (left eye)	83	M	Caucasian

**Table 2:** Basic sample information showing nuclei numbers and correlation with bulk RNA-seq of the same sample.

Donor	Region	Number of nuclei	Number of nuclei after QC	Correlation with bulk
12–887	Macular	1621	1428	0.68
12–887	Peripheral	415	388	0.66
13–0025	Macular	1613	1458	0.69
13–0025	Peripheral	424	391	0.67
13–0347	Macular	1805	1669	0.69
13–0347	Peripheral	664	539	0.71

Calculation of correlation was performed between the single-nuclei RNA-seq and bulk RNA-seq of the same samples. For single-nuclei RNA-seq (3′-end seq), the gene expression data from each nucleus were gene-filtered, normalized, and log-transformed (method) before averaging. For bulk RNA-seq (paired-end seq), FPKM was used

### Identification of major cell types in the human retina

**Fig. 2:**
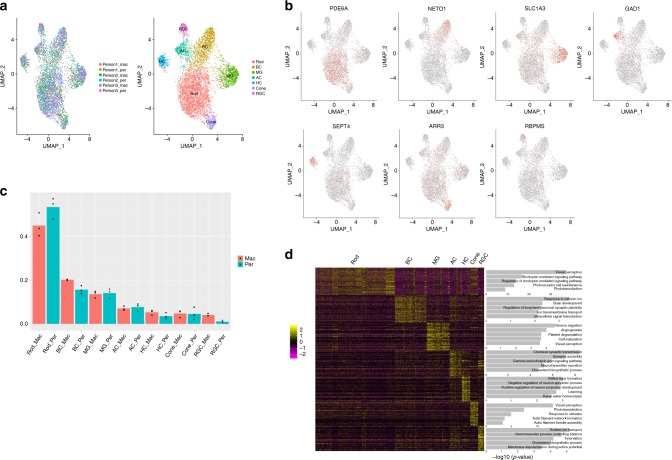
**Unsupervised clustering identifies seven major cell types in the human retina. a** Clustering of 5873 human retina single-nuclei expression profiles into seven populations (right) and representation of the alignment of six datasets from three donors (left). **b** Profiles of known markers (*PDE6A, NETO1, SLC1A3, GAD1, SEPT4, ARR3, RBPMS*) in each cluster. **c** The proportion of the seven cell types (rod, BC, MG, AC, HC, cone, RGC) in the macular and peripheral samples (bar graph shows the mean of the proportion; single data points are visualized in dots, *N* = 3). **d** Heatmap of DEGs in each cell type and the gene ontology term enrichment by each set of DEGs. For visualization, top 50 DEGs with least FDR q-value and top five terms under the biological process category with least *p*-value were used. Each column represents a cell while each row represents a gene. Gene expression values are scaled across all the cells.

To identify individual retinal cell types, we performed unbiased clustering on the gene expression profiles of 5873 human retinal nuclei. Seven clusters were identified, each of which contained cells from all six samples (Fig. 2a, Supplementary Fig. 1), suggesting relatively low sample bias. Based on the expression pattern of cell-type-specific marker genes in the cluster, each cluster is mapped to individual cell types (Supplementary Data 1). As shown in Fig. 2b, markers for known retinal cell types, such as *PDE6A* for rod cells, *NETO1* for bipolar cells (BC), *SLC1A3* for Müller glial cells (MG), *GAD1* for amacrine cells (AC), *SEPT4* for horizontal cells (HC), *ARR3* for cone cells and *RBPMS* for retinal ganglion cells (RGC), showed cluster-specific expression pattern. Thus, each cluster could be assigned to a known retinal cell type. Based on the number of nuclei in each cluster, we were able to quantify the proportion of each cell type in the sample. As shown in Fig. 2c and Table 3, the composition of different cell types from the human peripheral retina was generally consistent with that from previous mouse studies, with the exception of a higher percentage of MG cells and a lower percentage of AM cells observed in the human retina^10,16^, a piece of information that would require further experimental validation. This trend is consistent with the results reported from a previous study in monkey, in which the relative ratio of BC: MG: AC: HC is close to 40:28:22:9^16,17^. As expected, a lower rod proportion and higher BC, HC, and RGC proportions were observed in the human macular sample compared to the human peripheral retina. Furthermore, we noticed that the cone proportion in the macula region was only slightly higher than that of the peripheral, which was because that the macula samples collected for this study did not contain the fovea, where cone cells have a much more increased proportion. Since snRNA-seq is less biased in sampling in comparison to single-cell sequencing, a better estimation of cell proportion can be obtained. By comparing the transcriptome of cells in each cell type with all other cells, a total of 139, 101, 147, 167, 174, 255, and 249 cell type differentially expressed genes (DEGs) was identified for rod, BC, MG, AC, HC, cone, and RGC, respectively (here, DEGs are defined by transcriptome comparison between one cell type and all other cells, e.g., rods vs. non-rods; see methods; Supplementary Data 2). Gene ontology enrichment analysis of biological process terms was performed with these DEGs (Fig. 2d, Supplementary Data 3). Top GO terms enriched by each DEG lists were consistent with our previous knowledge for each cell type, such as visual perception term for photoreceptor cells^18^, ion transmembrane transport term for retinal interneurons^19–21^, and neuron migration term for Müller glia cells. These results indicated that our result faithfully represented the transcriptome profiles of major cell types of the human retina.

**Table 3:** Comparison of proportion of major cell types of mouse and human retina.

Cell type	Mouse retinal cell proportion (%) (Jeon et al.^16^)	Mouse retinal cell proportion (%) (Macosko et al.^10^)	Human macular cell proportion (%)	Human peripheral cell proportion (%)
Rod	79.9	65.6	44.81	53.29
Cone	2.1	4.2	4.81	4.61
MG	2.8	3.6	11.92	14.08
BC	7.3	14.0	20.12	15.60
AC	7.0	9.9	7.11	7.74
HC	0.5	0.6	5.33	3.61
RGC	0.5	1.0	4.15	1.07

### Regional gene expression variation revealed for human retina

**Fig. 3:**
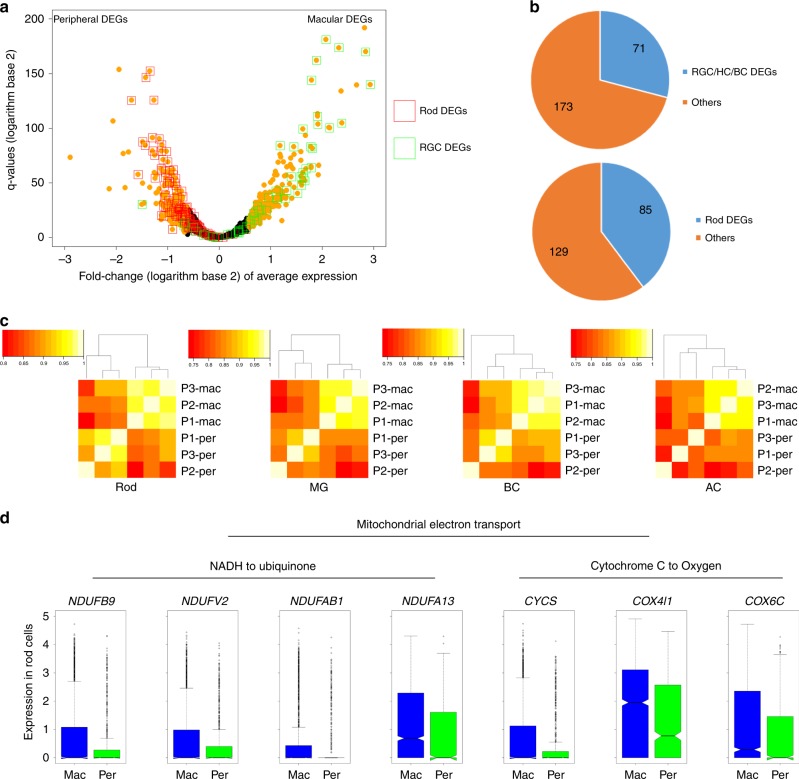
**Transcriptome difference revealed between macular and peripheral region. a** Differentially expressed genes between the overall macular and peripheral cells. All genes detected in more than 20% of macular or peripheral cells were plotted. Macular/Peripheral DEGs by overall comparison were labeled orange. Rod DEGs and RGC DEGs were labeled as red and green boxes, respectively. **b** The proportion of cell-type DEGs in the overall macular-peripheral DEGs showing cell population affection on DEG detection. **c** Heatmap demonstrating the correlation of averaged gene expression of four major cell types (rod, MG, BC, AC) in six samples. **d** Demonstration of mitochondrial-electron transport-related genes showing differential expression in rod cells in the macular and peripheral region. In the box plots, bounds of the box spans from 25 to 75% percentile, center line represents median, and whiskers visualize 1.5 times inter-quartile range less than quartile 1 or 1.5 times inter-quartile range more than quartile 3 of all the data points.

The macula is a structure unique to human and other primates. Several studies aimed at identifying genes that are differentially expressed between macular and peripheral retina have been conducted. For example, using a SAGE approach, Sharon et al. reported 20 genes with high expression in the macula and 23 genes with high expression in the peripheral region in the retina (referred as SAGE gene list)^22^. Based on bulk RNA-Seq, Li et al. reported 1239 genes with high expression in macular and 812 genes with high expression in the peripheral region^5^. To compare our data against these published data, we generated the virtual bulk macular and peripheral RNA-Seq data by in silico combining snRNA-Seq from each region. As a result, we obtained a list of 234 genes that were highly expressed in the macular region and 214 genes that were highly expressed in the peripheral region (Fig. 3a, Supplementary Data 4). Significant overlapping between the SAGE gene list and our list was observed. Specifically, for the SAGE gene list, 13 out of 21 macular genes (*SLC17A6, SNCG, NEFL, NEAT1, STMN2, YWHAH, UCHL1, DPYSL2, APP, NDRG4, TUBA1B, MDH1, EEF2*) and 11 out of 24 peripheral genes (*SAG, RCVRN, UNC119, GPX3, PDE6G, ROM1, ABCA4, DDC, PDE6B, GNB1, NRL*) were also observed in our gene list. However, most of the genes reported by the SAGE list but not included in ours (14 out of 21) also showed a consistent trend, with fold-change or expression proportion under our selected threshold. Similar GO terms, such as ion transport in macular genes and visual perception in peripheral genes (Supplementary Data 4), are enriched in both our dataset and the Li dataset (gene set not publicly available). Consistent data compared to previous studies further supports that our data are high quality and could be used for further analysis, such as macular-peripheral comparison within individual cell types.

In both the previous reports and our data, although the macular and the peripheral region consists of the same types of cell, the proportion of cell types are different between the macular and peripheral human retina (Fig. 2c, d). As a result, genes that exhibit different expression levels between the macular and peripheral regions in bulk transcriptome profiling experiments might be due to the differences in cell proportion instead of true expression level differences. In the 244 macular DEGs (‘virtual bulk’, compared with peripheral), 71 were also found in DEG lists that contained highly expressed genes of RGC, BC, or HC (comparison between the cell type with all other cells) (Fig. 3a, b). These cell types were found to be in a much higher proportion in the macular region (Fig. 2c, d). Consistently, out of 214 peripheral DEGs, 85 are found as rod DEGs (Fig. 3a, b), while rods were found to be in higher proportion in the peripheral region. These results suggested that the virtual bulk RNA-seq data tended to be affected by cell population variations in different regions in the retina and provided a general estimation of the limitations in the data found caused by small nuances in the population. Thus, single-cell level study is required for revealing genuine macular-peripheral similarities and differences. The snRNA-seq data overcome the constraint of the bulk RNA-seq data and allow a direct comparison of gene expression between macular and peripheral retina for the same cell type. Correlation of averaged gene expression patterns of four cell types (rod, MG, BC, and AC) from each sample were calculated (Fig. 3c). For all the four cell types, all samples showed reasonably high mutual correlation to each other, but the macula samples possessed a tighter cluster, indicating that differences do exist within the same cell type depending on the region. Within each cell types (rod, MG, BC, and AC) in the macular cells and peripheral cells, we performed differential expression analysis and found cell-type DEGs (Supplementary Data 4). Among the DEGs, it is interesting to note that some mitochondrial-electron-transport-related genes showed higher expression in macular rod cells compared to peripheral ones (Fig. 3d). Rod photoreceptor cells have large numbers of mitochondria packed in the inner segment^23^, because they require a higher amount of energy to maintain the high turnover rate of the outer segment and support phototransduction^24^. The comparatively higher expression level of these genes indicates higher oxidative stress, which is linked to photoreceptor death^25^. Mitochondria is a major source of retinal oxidative stress, which accumulates as the organism ages^26^. Mitochondrial dysfunction in retinal pigment epithelial cells (RPEs) has been associated with retinal diseases, like age-related macular degeneration (AMD), while its specific effect in photoreceptor cells remains relatively unknown^24^. Our finding might offer a potential explanation as to why macula photoreceptors are more vulnerable than peripheral ones. However, it is worth mentioning that the detected expression level of mitochondria-related genes could be affected by experimental conditions or sample biases. We confirmed that our findings were consistent with sample pairs from all three donors (Supplementary Fig. 2). In conclusion, the cell-type-based macular-peripheral comparisons reveal regional variances that bulk RNA-seq cannot find. Macular-peripheral differences within the same cell types were not studied with cone, HC, or RGC, because there was limited quantity of these cell types in the dataset.

### DEG analysis reveals human cone-rod differences

**Fig. 4:**
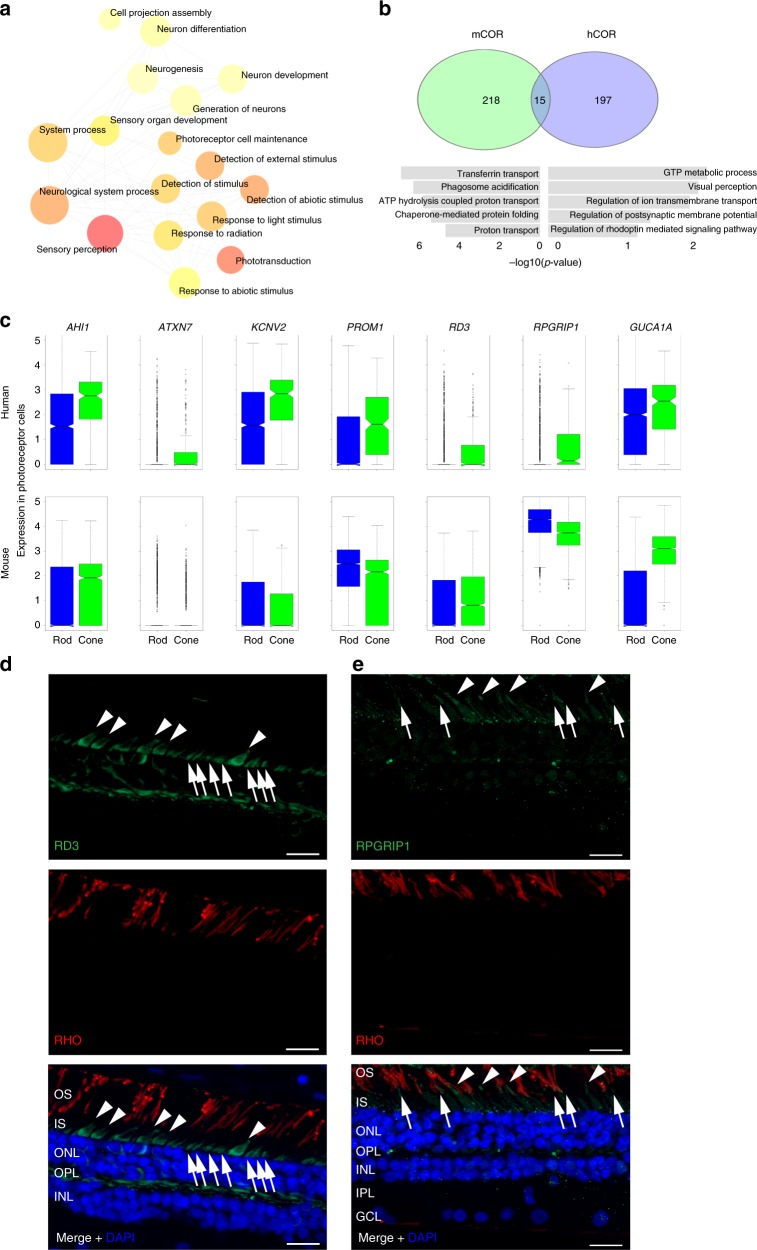
**Differentially expressed genes are revealed in rod and cone photoreceptors. a** Gene ontology networks demonstrate the biological process GO terms enrichment by photoreceptor cell DEGs. All photoreceptor cell DEGs (177 genes) were used as input for the online software NetworkAnalyst^76^ and only the terms with FDR adjusted *p*-value less than 0.05 were visualized. Terms were colored according to the *p*-value from low (red) to high (yellow). **b** Overlap of the hCOR (human cone-over-rod) and mCOR (mouse cone-over-rod) gene list and biological process GO term enriched in non-overlapping part. **c** Demonstration of the expression of CRD/LCA genes in the non-overlapping part of hCOR (*AHI1, ATXN7, KCNV2, PROM1, RD3, RPGRIP1*) and mCOR (*GUCA1A*). In the box plots, bounds of the box spans from 25 to 75% percentile, center line represents median, and whiskers visualize 1.5 times inter-quartile range less than quartile 1 or 1.5 times inter-quartile range more than quartile 3 of all the data points. **d** Immunofluorescent staining for RD3 and RHO on human retina cryosections. White triangles and arrows highlight the fluorescent signal of the cones and rods, respectively, in the top and bottom panel. Retinal cell layers are labeled in the bottom panel (OS outer segment, IS inner segment, ONL outer nuclear layer, OPL outer plexiform layer, INL inner nuclear layer, IPL inner plexiform layer, GCL ganglion cell layer). Scale bar is 20μm. **e** Immunofluorescent staining for RPGRIP1 and RHO on human retina cryosections. White triangles and arrows highlight the fluorescent signal of the cones and rods, respectively, in the top and bottom panel. Retinal cell layers are labeled in the bottom panel. Scale bar is 20μm.

More than half of the retina cell population is composed of photoreceptor cells. By combining rod and cone photoreceptor cells, a list of 177 genes that are highly expressed in photoreceptors was obtained (PR cells compared with all other cells, Supplementary Data 2). These genes show significant enrichment of biological process GO terms of phototransduction, sensory perception, response to light stimulus, etc. (Fig. 4a). This is mostly consistent with the known functions of photoreceptor cells, which further validate our cluster assignment and DEG analysis.

In photoreceptor cells, cones are important for function^1,18,27,28^ but poorly studied. During retina development, photoreceptor precursors first commit to their photoreceptor cell fate and then differentiate into subtypes, namely rods and cones^29^. Thus, cone cells and rod cells are closely related in development and share similarities in functions. Comparison of transcriptomes between cone cells and rod cells would provide informative insights to cone-specific functions. Genes with higher cone expression compared to rod expression may be relevant in determining cone-specific functions. We identified 212 genes that were highly expressed in human cone cells compared to rod cells (human cone-over-rod gene list, referred to as hCOR list, Supplementary Data 5, Supplementary Fig. 3). GO analysis on biological process terms revealed that these genes enrich terms such as visual perception, GTP metabolic process, phototransduction, response to stimulus, and regulation of ion transmembrane transport (Supplementary Data 5). Using published single-cell RNA-seq data of mouse retina^10^, rod and cone cells were identified with a method similar to what is used for human data. Two hundred thirty three genes that were highly expressed in cone cells compared to rod cells were identified (mouse cone-over-rod list, referred to as mCOR list, Supplementary Data 6). Interestingly, this mCOR list shared less than 10% similarity to the hCOR list (Fig. 4b), indicating considerable differences between human and mouse cone cells. Excluding the 15 shared genes by hCOR and mCOR, the rest of the genes in hCOR enrich biological process GO terms, such as GTP metabolic processes, visual perception, and regulation of ion transmembrane transport, while the top terms enriched by the mCOR list were not phototransduction-related. This result indicated more insights of cone cell functions could be potentially obtained within our dataset. Indeed, in the hCOR list (excluding shared genes), 7 genes (*AHI1, ATXN7, KCNV2, PROM1, RD3, RPGRIP1*) have been linked to human CRD or Leber’s congenital amaurosis (LCA) diseases, while mCOR list only contains one such gene (*GUCA1A*). As an example, *RPGRIP1* and *RD3*, both are known human IRD genes^30–39^, are expressed at a significantly higher level in human cones compared to rods, as is shown in Fig. 4c. Consistent with the expression pattern found, patients with mutations in *RPGRIP1* and *RD3* show LCA and CRD phenotype, where more severe defects are found in cones than rods. In contrast, these two genes show no differential expression in rod and cone cells in the mice dataset (Fig. 4c). In live animals, KO mouse models of these two genetic defects are reported to display retinitis pigmentosa (RP)-like phenotypes^39–41^, which are the result of early defects in rod cells. With immunofluorescence staining, we confirmed our findings that the expression level of RPGRIP1 and RD3 was higher in human cone cells compared to human rod cells (Fig. 4d, e). Additionally, the expression pattern of RPGRIP1 in macaque photoreceptor cells reported by Peng et al.^42^ is consistent with our finding (RD3 was not well detected in the macaque data, Supplementary Fig. 4). Therefore, the differences in mouse and human phenotype are at least partially due to differences in cell-specific expression of the gene. The human cone profile would serve as an informative resource to better understand mechanisms behind human retinal biology and diseases.

### Retinal disease genes are enriched in photoreceptor DEGs

**Fig. 5:**
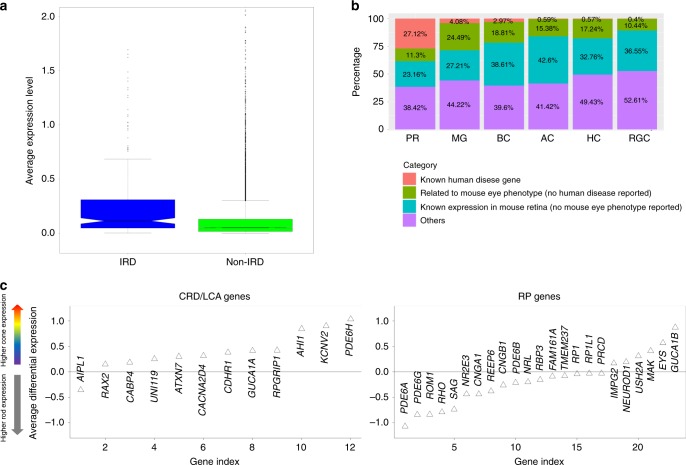
**Photoreceptor DEGs enrich human IRDs. a** IRD genes generally show higher expression level than the rest of genes in the human retina. Expression values for each gene were normalized by cell total reads, multiplied by 10,000 and then log-transformed (natural logarithm), before averaging. In the box plots, bounds of the box spans from 25 to 75% percentile, center line represents median, and whiskers visualize 1.5 times inter-quartile range less than quartile 1 or 1.5 times inter-quartile range more than quartile 3 of all the data points. **b** Distribution of human retinal disease genes, mouse eye phenotype genes (but no human disease discovered), mouse retinal expressing genes (but no eye phenotype found) in DEGs of all cell types. **c** RP genes and CRD genes show different expression trend in rod and cone photoreceptors. The *y*-axis is representing the differential expression of each gene in cone cells compared with rod cells (cone expression level minus rod expression level). Expression values for each gene were normalized by cell total reads, multiplied by 10,000 and then log-transformed (natural logarithm), before averaging within cell types (rod, cone).

With the expression profile for each retinal cell type generated in this study, we sought to examine its potential utility in identifying genes associated with human retinal diseases. A gene list of 246 genes that include known IRD genes for retinitis pigmentosa (RP), Leber’s congenital amaurosis (LCA), cone-rod dystrophy (CRD), and other retinopathies was obtained from the retnet (RetNet, http://www.sph.uth.tmc.edu/RetNet/) (Supplementary Data 7). As expected, robust expression of most of these known IRD genes (233 of the 246) could be detected in our dataset (Supplementary Data 7). Additionally, the detected IRD genes were expressed at a significantly higher level than the average (Fig. 5a, *p*-value = 1.22e-08, two-sample *t*-test). Since the vast majority of known IRD-associated genes affects photoreceptor cells exclusively, we believed that significant overlap should be detected between the IRD genes and the photoreceptor-enriched gene set. We investigated the PR DEG list (Supplementary Data 2), which includes 177 genes that exhibit significantly higher expression in photoreceptor cells than other cell types (may not be rigorously exclusive). Indeed, 48 IRD genes are among the 177 PR DEGs, representing a significant enrichment over background (odds ratio = 27.06, *p*-value < 2.2e-16, fisher’s exact test) (Fig. 5b, Supplementary Data 8). This result outperforms previous reports using bulk RNA-seq data. Pinelli et al. generated an RNA-seq of 50 retina samples and used co-expression analysis to predict potential IRDs^4^ and only 56 known retinal disease associated genes are obtained from a list of 472 genes, an odds ratio of 15^4^. Due to its higher performance quality, we propose that the PR DEG list can be potentially used as a gene prioritization tool for novel IRD gene discovery.

Depending on the timing and severity of rod and cone photoreceptors defects, IRDs can be classified into different subtypes clinically. For example, although cone photoreceptor cell degeneration is observed as the disease progresses, RP is primarily due to a defect in rod photoreceptors^43–45^. In contrast, CRD and LCA mostly result from cone degeneration from the very beginning^8,9^. Among the 48 IRD genes that show photoreceptor cell-specific expression, 23 have been associated with RP, while 12 have been associated with CRD or LCA. This is consistent with the clinical phenotype where, on average, RP-associated genes have higher expression in rod cells while CRD- or LCA-associated genes show higher in cone cells (Fig. 5c). It is worth noting that six of the 23 RP genes actually showed higher expression in cone cells, which are ‘counter examples’. This indicates that the tolerance of malfunction of some gene products by rods and cones- though the products are required by both cell types- could be different. One possible reason could be that the difference of the rod and cone transcriptome could lead to different redundant features.

Given the significant enrichment of IRD-associated genes in the cell-type DEG set, it can be potentially used to prioritize candidate IRD disease genes. For the 129 photoreceptor DEGs that have yet to be associated with human retinal diseases, 61 has already shown expression in mouse retina (MGI gene expression data). Furthermore, viable knock-out mice have already been obtained for 93 genes. Among these 93 genes, phenotypes in the visual system have been observed in 20 genes (MGI database; Supplementary Data 8), such as *GNGT1*, *RDH8*, and *RCVRN*. The protein encoded by *GNGT1* is known to be located in the outer segment of photoreceptor cells and plays an important role in phototransduction^46,47^. *RDH8* gene encodes a human retinol dehydrogenase, and a mutation of its mouse ortholog causes delayed rod function recovery after light exposure and an accumulation of all-trans-retinal in the rod outer segment^48^. *RCVRN* is a calcium-binding protein, a regulator of rod sensitivity of dim lights, and it is also found to be an auto-antigen of cancer-associated retinopathy^49^. Although this list proves itself useful, the power of this list for prioritizing candidate IRD genes could be underestimated because some genes could have mutations that would cause an eye phenotype in mice but might not be reflected in the MGI data. For example, *Top2b* conditional knock-out mice have been reported to show severe photoreceptor cell loss during eye development^50^; however, this phenotype was not captured by the MGI data. In summary, our photoreceptor cell DEG list would serve as a useful prioritization tool for novel diseased gene discovery.

### Retinal disease genes in other retinal cell types

In contrast to the significant overlap between IRD genes and photoreceptor (PR) DEGs, no significant enrichment is observed in DEGs for other retinal cell types. Although rare, it has been shown that defects in cell types in the neural retina other than PR can also lead to IRD. Indeed, 12 known IRD genes show enriched expression in cell types not restricted to photoreceptor cells (Supplementary Data 8). For example, *RDH11* is highly expressed in Müller Glia cell, which plays an important function in the retinoid cycle, and mutations in *RDH11* lead to RP^51^. *GRM6* and *TRPM1* are bipolar DEGs and their mutations could cause a recessive congenital stationary night blindness (CSNB), a condition due to abnormal photoreceptor-bipolar signaling^52–58^. In addition, 36, 30, 26, 26, and 19 genes that are highly expressed in MG, HC, RGC, AC, and BC, respectively, showed mouse eye phenotypes (Supplementary Data 8). These genes are likely associated with human diseases.

## Discussion

Studying the cell-type specific transcriptome expands our understanding of the cell function within heterogeneous tissues, including the retina. The retina contains seven major cell types in differing proportions: rod cells consist of over half while other types, such as amacrine cells and RGCs, are much rare. All the cell types have distinct functions and coordinate to allow for visual perception and regulation. Under pathological conditions, not all the cell types are affected equally at the early stage of cell development^8,43,59^. Thus, understanding the transcriptome at the cell type or the single-cell level will expand disease-related studies. In the retina, transcriptome profiles of many cell subtypes, especially rare ones, are usually masked in bulk RNA-seq. Cell-surface-marker-based sorting and purification methods have not been developed to enrich all cell types. Thus, single-cell RNA-seq stands out as the most effective and unbiased method for obtaining the transcriptome of each cell types in the retina.

Efforts have been made to obtain single-cell level transcriptome profiles of human or primate retina^42,60^. Here, we report a transcriptome profiling of the human retinal major cell types at the single-cell level from 5873 nuclei with the snRNA-seq method. These nuclei were from six samples, obtained from three donor retinae. It is worth emphasizing that the donors had a similar genetic background, all underwent post-mortem phenotyping, and were confirmed to has normal retina. OCT images are used to resolve the appearance of subretinal drusen, fluid, atrophy, and fibrosis, and to differentiate artifact from pathology. These images (Fig. 1) demonstrate the usage of a combination of fundus and OCT imaging techniques for examining post-mortem eye common in clinical practice and in accordance to the Utah Grading Scale for Post-mortem Eyes^61^. All major cell types were found in every sample within a reasonable proportion and expected marker expression. Thus, the transcriptome profiles we demonstrated here are reproducible and reliable.

The use of snRNA-seq method especially ensured the quality of our dataset, as the RNA integrity of the samples could be well preserved with a much-reduced death-to-preservation time compared to other methods that used cell dissociation. Another advantage of the snRNA-seq is that the sampling bias could be minimized, which is an issue for profiling complex tissue. Thus, our estimation of cell proportion is likely to be more accurate compared to previous results. In terms of faithfully representing the transcriptome, Gao et al. reported that, in breast cancer cells, the snRNA-seq could be representative of single-cell RNA-seq^62^. To corroborate these previous findings, our single-nuclei profiles showed a reliable correlation to bulk RNA-seq of the same sample, and the cell-type profiles are also consistent with published human retinal cell markers.

Regional transcriptomes for tissues are of great interest for researchers while single-cell level studies could provide insight to future research questions. Here, we demonstrated that, for heterogeneous tissue, single-cell studies out-perform bulk studies in regional specific transcriptome profiling. We concluded that bulk studies are limited by variations of the cell population in different regions, where findings with variable expression levels in genes may be due to changes in cell proportions, rather than true changes in expression in genes. Single-cell studies, on the other hand, allows for comparisons between each cell types to eliminate this source of error. Interestingly, based on our dataset, it is observed that genes related to mitochondrial-electron transport showed higher expression in macular rod cells compared to peripheral ones, suggesting that the macular rod cells may have higher oxidative stress. In a recent article, Voigt et al.^63^ reported a dataset of human retina using single-cell RNA-seq and identified genes that showed differential expression when comparing the corresponding cell type from the foveal and peripheral retina. By comparing our results to the Viogt dataset, we found that these two datasets are largely consistent. For example, among the top 20 DEGs between foveal and peripheral cones in the Voigt dataset, 17 were detected in our dataset, with 10 showing consistent trend with both the ‘Voigt dataset’ and the dataset reported by Peng et al.^42^ (Supplementary Fig. 5).

The transcriptome profiles of all major cell types, especially cone cells, in the human retina are also important findings in our study. Mice, the most studied animal model for retinal degeneration, are not an ideal model for studying cone biology since its cones are significant different from those in humans. For example, in the human retina, cone cells are highly rich in a region called the fovea near the central macula, which is absent in the mouse retina^13,64^. In addition, mice have two types of cone-opsins, namely *Opn1sw* for short-wavelength sensing and *Opn1mw* for middle- and long-wavelength sensing. Some mouse cone cells express only one type of opsin but a considerable proportion (40% for C57/BL6 mice) express both^65^. In contrast, humans have three types of cone-opsins, *OPN1SW*, *OPN1MW*, and *OPN1LW*, and each cone cell only expresses one type of opsin^29^. Mustafi et al. previously compared the transcriptomes between the cone-enriched macular region and rod-enriched peripheral region in monkey to analyze rod and cone signatures^7^. They found known cone makers, such as *SLC24A2* and *OPN1MW*; however, they also found other genes, including *NEFL* and *NEFM*, which were actually highly expressed in RGCs. This finding had limited sensitivity and might be driven by the uneven proportion of RGCs. Welby et al. developed a sorting method to specifically select fetal cones in human and reported the transcriptome of these fetal cone cells^6^. Their findings identified the cone signature during the development (9–20 weeks post conception), which could be useful in recapitulating some aspects of the human adult cone. Our study took advantage of in silico sorting to separate cones from other cell types and reported the human cone cell transcriptome profile, which could be a useful resource in future studies. With this cone profile, we were able to perform a cone-rod comparison to gain insight into cone function and investigate discrepancies between phenotype in patients and phenotype of genetic mouse models of cone diseases.

The rich single-cell transcriptome profiles could be a useful resource for the research community. For example, we observed significant enrichment of IRD genes among the genes with high specificity in photoreceptor cells. In addition, the list also contains multiple genes that lead to eye defect in mice when knocked out. Thus, we could use this list to prioritize novel IRD gene discovery. Additionally, it is worth noting that a list of genes specifically expressed in cells other than PR was also found to cause retinal phenotype in human and/or mice when mutated. Besides the uses that were described, this dataset can be informative for many other studies. For instance, the cell-type-specific markers reported in our data would facilitate general cell-type-specific studies.

As a proof of concept study, this dataset has certain limitations. The complexity of the retina could not be fully solved with the number of nuclei we sequenced. For example, due to the modest number of nuclei profiled in this study, this dataset is underpowered and cannot be used to classify subtypes of cells in the retina. Recognizing this limitation, we mainly focused on the profiles of the major cell types in the retina in this study. Nevertheless, given the robustness of snRNA-Seq, it is feasible to scale up the current study to provide much improved resolution in future studies.

## Methods

### Macular and peripheral sample collection

As previously described in detail^61^, human donor eyes were obtained in collaboration with the Utah Lions Eye Bank. Only eyes within 6 h post-mortem were used for this study. Both eyes of the donor underwent rigorous post-mortem phenotyping, including spectral domain optical coherence tomography (SD-OCT) and color fundus photography. Most importantly, these images were taken in a manner consistent with the appearance of the analogous images utilized in the clinical setting. Dissections of donor’s eyes were carried out immediately according to a standardized protocol to reliably isolate the RPE/choroid from the retina and segregate the layers into quadrants^61,66^. After the eye was flowered and all imaging was complete, macula retina tissue was collected using a 6 mm disposable biopsy punch (Integra, Cat # 33–37) centered over the fovea and flash frozen and stored at −80 °C. The peripheral retina was collected in a similar manner for each of the four quadrants. To determine precise ocular phenotype relative to disease and healthy aging, analysis of each set of images was performed by a team of retinal specialists and ophthalmologists at the University of Utah School of Medicine, Moran Eye Center and the Massachusetts Eye and Ear Infirmary Retina Service. Specifically, each donor’s eye was checked by an independent review of the color fundus and OCT imaging; discrepancies were resolved by collaboration between a minimum of three specialists to ensure a robust and rigorous phenotypic analysis. This diagnosis was then compared to medical records and a standardized epidemiological questionnaire for the donor. For this study, both eyes for each donor were classified as AREDS 0/1 to be considered normal. Only one eye was used for each donor. Donors with any history of retinal degeneration, diabetes, macular degeneration, or drusen were not used for this study. Institutional approval for the consent of patients to donate their eyes was obtained from the University of Utah and conformed to the tenets of the Declaration of Helsinki. All retinal tissues were de-identified in accordance with HIPAA Privacy Rules.

### Preparation of single-nucleus suspensions

Nuclei from frozen neural retinal tissue was isolated using RNase-free lysis buffer (10 mM Tris-HCl, 10 mM NaCl, 3 mM MgCl_2_, 0.1% NP40). The frozen tissue was resuspended in ice-cold lysis buffer and triturated to break the tissue structure. The tissue aggregates were then homogenized using a Wheaton™ Dounce Tissue Grinder and centrifuged (500 g) to pellet the nuclei. The pellet was resuspended in fresh lysis buffer and homogenized to yield clean single-nuclei suspension. The collected nuclei were stained with DAPI (4′,6-diamidino-2-phenylindole, 10 ug/ml) and were diluted to 1000 μl of 3E4/ml with 1 × PBS (without Ca and Mg ions, pH 7.4, Thermo Fisher), RNase inhibitor (NEB, 40 KU/ml) and Cell Diluent Buffer.

### The ICELL8™ single-cell-based single-cell capture

Single nuclear capture and sequencing were performed on the ICELL8 single-cell platform (Wafergen Biosytems). ICELL8 platform comprised of a multi-sample nano-dispenser that precisely dispensed 50 nl of the single-nuclei suspension into an ICELL8 nanowell microchip-containing 5184 wells (150 nl capacity). Assuming a Poisson distribution frequency for the number of cells per well, about 30% of the nanowells were expected to contain a single nucleus under optimal conditions. Automated scanning fluorescent microscopy of the microchip was performed using an Olympus BX43 fluorescent microscope with a robotic stage to visualize wells containing single nuclei (see Table 1 for single-cell capture number across different experimental repeats). The automated well selection was performed using the CellSelect software (Wafergen Biosystems), which identified nanowells containing single nuclei and excluded wells with >1 nuclear, debris, nuclei clumps or empty wells. The candidate wells were manually evaluated for debris or clumps as an additional QC.

### Single-cell RT-PCR and library preparation

The chip was subjected to freeze-thaw in order to lyse the cells and 50 nl of reverse transcription and amplification solution (following ICELL8 protocol) was dispensed using the multi-sample nano-dispenser to candidate wells. Each well had a preprinted primer that contains an 11-nucleotide-well-specific barcode. This barcode was added to the 3′ (A)n RNA tail of the nuclear transcripts during reverse transcription and cDNA amplification, the latter of which was performed on candidate wells using SCRB-seq chemistry. After RT, the cDNA products from candidate wells were pooled, concentrated (Zymo Clean and Concentrator kit, Zymogen) and purified using 0.6× AMPure XP beads. A 3′ transcriptome enriched library was made using Nextera XT Kit and 3′-specific P5 primer, which was sequenced on the Illumina Hiseq2500.

### Immunofluorescence (IF)

Healthy human donor retinal tissue was dissected and fixed in 4% PFA for 48 h, cryo-protected with 30% sucrose overnight in 4 degrees, and then embedded in OCT to be flash-frozen. Cryosections from a peripheral region of the human retina with 10 μm thickness were used for the IF.

For the IF, sections were fixed with 4% PFA for 5 min at room temperature. They were then blocked for 3 h with blocking buffer (10% normal goat serum in PBS + 0.1% Triton X-100) in room temperature. Sections were then incubated overnight at 4 °C with primary antibodies (anti-RD3: Thermo Scientific PA583118; anti-RPGRIP1: gift from Dr. Tiansen Li’s lab; anti-RHO 1D4: Santa Cruz 57432) diluted in blocking buffer (1:150 for anti-RD3 and anti-RHO 1D4; 1:50 for anti-RPGRIP1). Species-specific fluorophore conjugated secondary antibody in blocking buffer (1:200) was applied for 120 min in room temperature, followed by DAPI staining for 10 min. The sections were washed with PBS (three times, 5 min each) between every buffer change. The sections were then mounted with AquaMount Slide Mounting Media (Thermo Scientific 13800).

### Data analysis

Generation of human snRNA-seq expression matrices and quality control. FASTQ files were generated from Illumina base call files using bcl2fastq2 conversion software (v2.17). Sequence reads were aligned to the human genome hg19 (GRCh37), and aligned reads were counted within exons using HTseq-count using default parameters^67^ to generate the expression matrices of raw read counts. Quality control of the expression matrices of six samples (macular region and peripheral region for each of the three tissues) were performed separately before they were merged together. For quality control of each matrix, genes that were not detected in at least 0.5% of all cells were discarded. Cells were filtered based on a minimum number of 500 and a maximum number of 3000 expressed genes per cell, and a minimum number of 6000 and a maximum number of 100000 transcripts per cell.

Data alignment and unsupervised clustering. For all six expression matrices, the expression value of each gene in each cell was normalized by the following conversions: total read counts of that cell multiplied by 10,000 and then log-transformed (natural logarithm, ln (value + 1)). Using the Seurat v3 package^68^, variable genes were detected in each matrix and were used as input for the ‘FindIntegrationAnchors’ function, and thus the six matrices were integrated with the ‘IntegrateData’ function. The integrated data were then clustered with principal component analysis (PCA; top 10 principal components were used) and the clusters were visualized in two dimensions with UMAP. All the data alignment, integration, and clustering were performed under standard Seurat workflow.

Detection of differentially expressed genes (DEGs) for each cluster. Raw read count was used as input for DEG analysis with the VGAM R package implemented by the Monocle 3 R package^69^. For obtaining the DEG list of each cluster, only the genes expressed in more than 20% of that cluster were taken. For each gene, the expression level of all other clusters was used as background for comparison. DEGs were defined as genes that had a 1.5-fold expression over the background with a q-value (FDR) less than 0.05. For calculating the fold-change, we used the raw read counts normalized with total read counts in each cell and averaged by cluster (or background). DEGs between two clusters were obtained in similar methods, where all the genes with more than 20% detection in either cluster were used. With the gene lists, functional enrichment analysis was performed using DAVID^70^.

For bulk RNA-seq on the same samples, FASTQ sequences were mapped to human genome hg19 (GRCh37), which was downloaded from UCSC genome browser website and aligned using STAR^71^. Transcript structure and abundance were estimated using Cufflinks^72–75^.

For mouse single-cell RNA-seq, the expression data was obtained from GSE63473^10^. Matrices from seven P14 mice, GSM1626793-1626799, were used for analysis. The major analysis pipeline, including data normalization, integration, clustering, and DEG detection, was the same as the one used for human data. The only differences were in data QC. For mice data, cells were filtered based on a minimum number of 400 and a maximum number of 3000 expressed genes per cell, and a minimum number of 800 transcripts per cell.

## Supplementary information

Supplementary Information

Description of Additional Supplementary Files

Supplementary Data 1

Supplementary Data 2

Supplementary Data 3

Supplementary Data 4

Supplementary Data 5

Supplementary Data 6

Supplementary Data 7

Supplementary Data 8

## Data Availability

All relevant data are available from the authors. snRNA-seq data could be accessed by GEO series number GSE133707.

## Code availability

The Seurat v3 and monocle 3 R packages are major tools for the single-cell RNA-seq data analysis and standard workflows are used. All relevant code is available from the authors.
