# Supplementary material for: Single-nuclei RNA-seq on human retinal tissue provides improved transcriptome profiling
Source: Nat Commun. 2019 Dec 17;10:5743. doi: 10.1038/s41467-019-12917-9 (PMC6917696; doi:10.1038/s41467-019-12917-9)
Supplement: Supplementary file 1 — Supplementary Information [file 41467_2019_12917_MOESM1_ESM.pdf]

**Single-nuclei RNA-seq on human retinal tissue provides improved transcriptome profiling**

Qingnan Liang[1, 2, 3, 8] , Rachayata Dharmat[1, 8], Leah Owen[4], Akbar Shakoor[4], Yumei Li[1], Sangbae Kim[1], Albert Vitale[4], Ivana Kim[4], Denise Morgan[4, 5], Shaoheng Liang[7], Nathaniel Wu[1], Ken Chen[7], Margaret M DeAngelis[4, 5, 6]#, Rui Chen[1, 3]#

# Corresponding authors. Margaret M DeAngelis, margaret.deangelis@utah.edu; Rui Chen, ruichen@bcm.edu

[1]HGSC, Department of Molecular and Human Genetics, Baylor College of Medicine, Houston, TX, 77030, United States.

[2]Biochemistry and Molecular Biology Graduate Program, Baylor College of Medicine, Houston, TX, 77030, United States.

[3]Verna and Marrs McLean Department of Biochemistry and Molecular Biology, Baylor College of Medicine, Houston, TX, 77030, United States.

[4]Department of Ophthalmology and Visual Sciences, University of Utah School of Medicine, Salt Lake City, UT, 84132, United States.

[5]Department of Pharmacotherapy, the College of Pharmacy, University of Utah, Salt Lake City, UT, 84132, United States.

[6]Department of Population Health Sciences, University of Utah School of Medicine, Salt Lake City, UT, 84132, United States.

[7]Department of Bioinformatics and Computational Biology. The University of Texas MD Anderson Cancer Center, Houston, TX, 77030, United States.

[8]These authors contribute equally to this work.

## Supplementary Figures

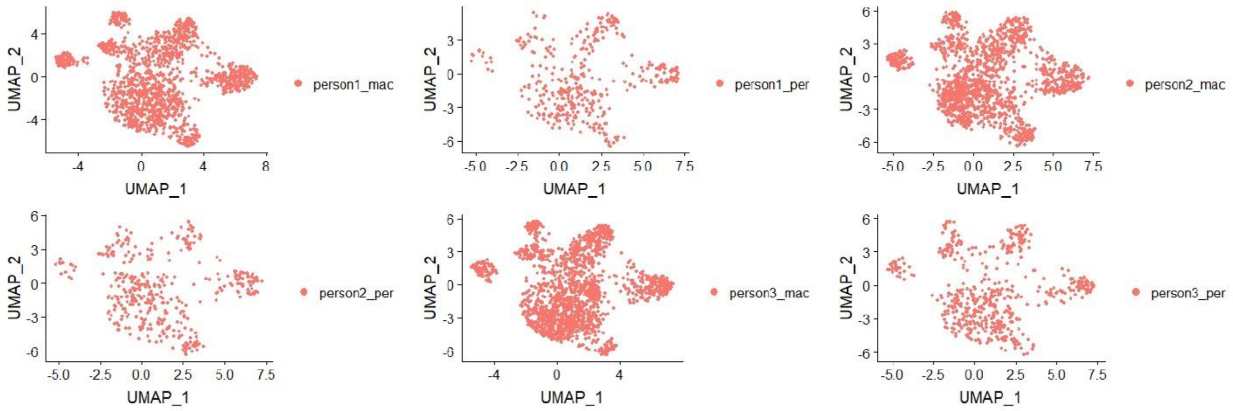


**Supplementary Figure 1. Visualization of cluster distribution of each sample**

Projection of cells from each sample to 2-D space showing how each sample distribute to each cluster.

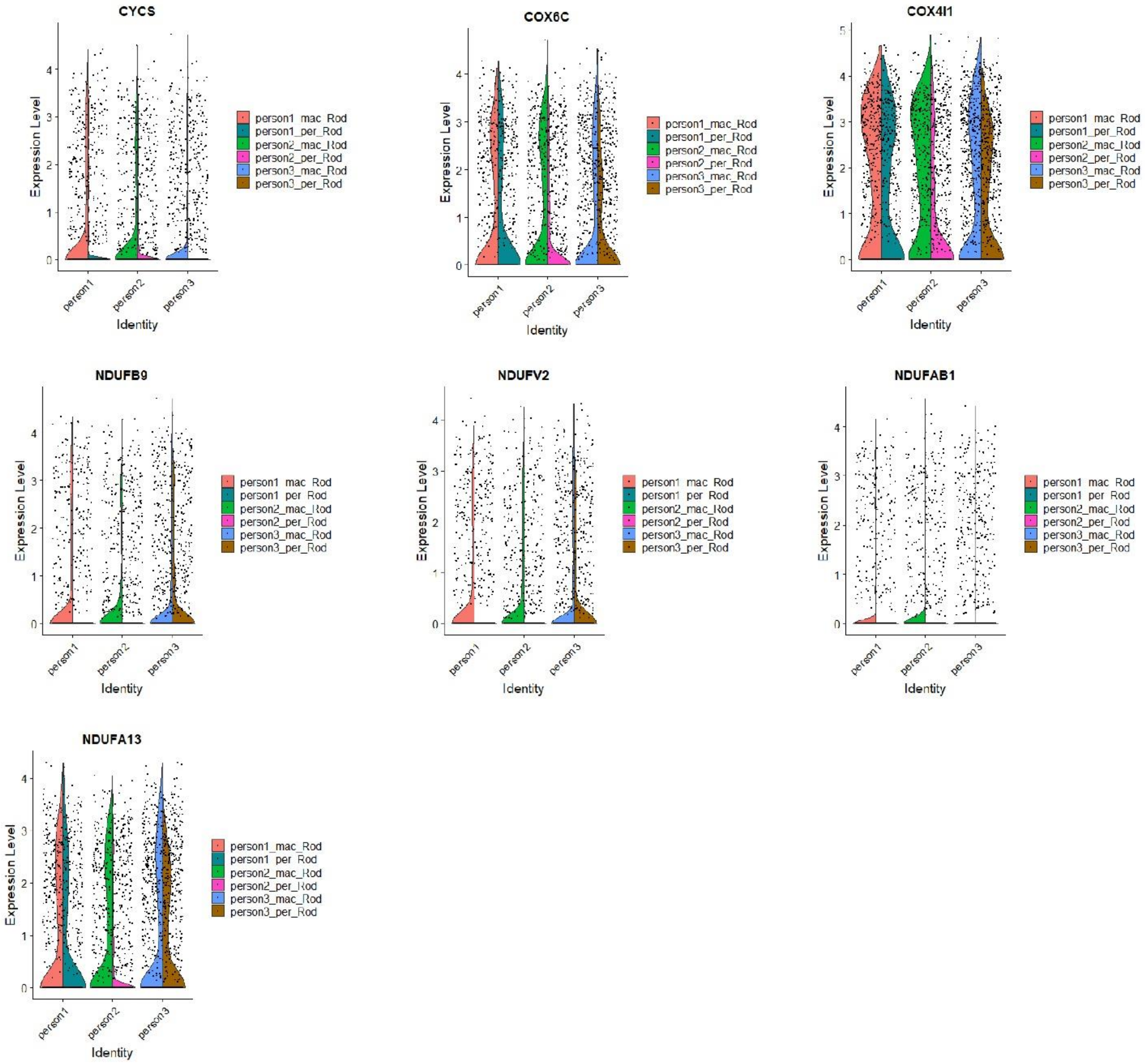


**Supplementary Figure 2. Expression of mitochondrial electron transport genes in rod cells across allsamples.**

Violin plot showing the expression level (normalized by cell total reads, multiplied by 10,000 and then log transformed (natural logarithm)) of seven mitochondrial electron transport related genes which had higher expression in macular rods.

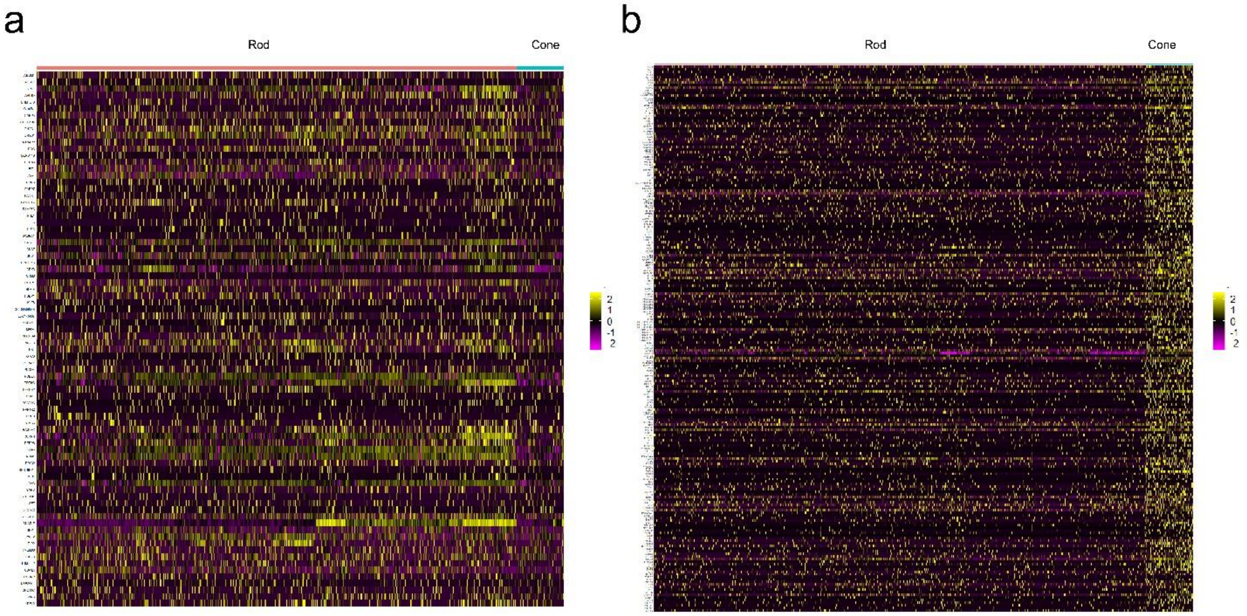


**Supplementary Figure 3. Visualization of human rod-cone differentially expressed genes**

a. Heatmap of rod-cone DEGs that are highly expressed in rods. Each column represents a cell while each row represents a gene. Gene expression values are scaled across all the photoreceptor cells.

b. Heatmap of rod-cone DEGs that are highly expressed in cones. Each column represents a cell while each row represents a gene. Gene expression values are scaled across all the photoreceptor cells.

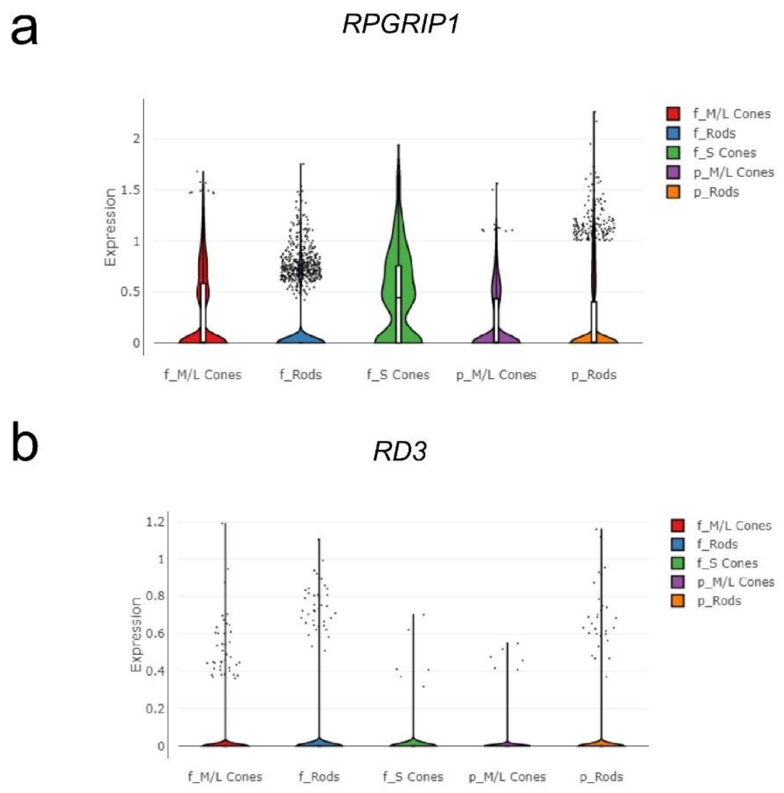


**Supplementary Figure 4. Expression level of RPGRIP1 and RD3 in *macaca fascicularis* photoreceptor cells*.***

a. Violin plot of the expression level of the *RPGRIP1* gene in macaca fascicularis photoreceptor cells. Data of foveal M/L-cones, foveal rods, foveal S-cones, peripheral M/L-cones, and peripheral rods (left to right) are shown in red, blue, green, purple and orange, respectively. Figure was made from https://portals.broadinstitute.org/single_cell.

b. Violin plot of the expression level of the *RD3* gene in macaca fascicularis photoreceptor cells. Data of foveal M/L-cones, foveal rods, foveal S-cones, peripheral M/L-cones, and peripheral rods (left to right) are shown in red, blue, green, purple and orange, respectively. Figure was made from https://portals.broadinstitute.org/single_cell.

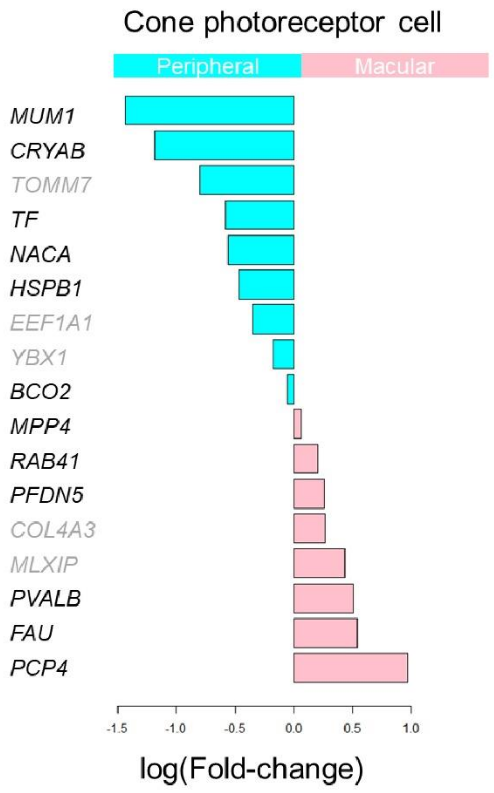


**Supplementary Figure 5. Macular and peripheral expression of genes reported as foveal/peripheral cone DEGs by Voigt et al[1].**

Fold-change of the 17 genes (among the top 20 reported by Voigt et al) expression level in macula and peripheral cone cells. Genes with consistent spatial expression trend with the dataset of Voigt et al[1] or Peng et al[2] are labelled black. Genes with inconsistent spatial expression trend were labelled grey.

**References:**


1. Voigt, A. P. *et al.* Molecular characterization of foveal versus peripheral human retina by single-cell RNA sequencing. *Exp. Eye Res.* **184,** 234–242 (2019).

2. Peng, Y.-R. *et al.* Molecular Classification and Comparative Taxonomics of Foveal and Peripheral Cells in Primate Retina. *Cell* **176,** 1222–1237.e22 (2019).
