# Supplementary material for: Single-nuclei RNA-seq on human retinal tissue provides improved transcriptome profiling
Source: Nat Commun. 2019 Dec 17;10:5743. doi: 10.1038/s41467-019-12917-9 (PMC6917696; doi:10.1038/s41467-019-12917-9)
Supplement: Supplementary file 2 — Description of Additional Supplementary Files [file 41467_2019_12917_MOESM2_ESM.docx]

**Description of Additional Supplementary Files**

Supplementary Data 1. Marker genes used for assignment of major cell types to the clusters.

Supplementary Data 2. Differentially expressed genes for each cell type, compared with all the other cells.

Supplementary Data 3. Enrichment of gene ontology terms (biological process) in the differentially expressed genes of each cell type.

Supplementary Data 4. Differentially expressed genes between the macular and peripheral region of the retina.

Supplementary Data 5. Differentially expressed genes between rod cells and cone cells, and the enrichment of gene ontology terms (biological process) of these genes.

Supplementary Data 6. Gene lists containing non-overlapping cone-over-rod genes in human and mouse single-cell dataset and the enrichment of gene ontology terms (biological process) of these genes.

Supplementary Data 7. Disease related genes used in this current study.

Supplementary Data 8. Annotation of differentially expressed genes in each cell type by disease relevance, mouse eye phenotype and mouse retinal expression.
